# Development and preliminary validation of a Family Nutrition and Physical Activity (FNPA) screening tool

**DOI:** 10.1186/1479-5868-6-14

**Published:** 2009-03-12

**Authors:** Michelle A Ihmels, Greg J Welk, Joey C Eisenmann, Sarah M Nusser

**Affiliations:** 1 Department of Kinesiology, Iowa State University, Ames, Iowa, USA; 2 Department of Kinesiology, Michigan State University, East Lansing, Michigan, USA; 3 Department of Statistics, Iowa State University, Ames, Iowa, USA

## Abstract

**Background:**

Parents directly influence children's physical activity and nutrition behaviors and also dictate the physical and social environments that are available to their children. This paper summarizes the development of an easy to use screening tool (The Family Nutrition and Physical Activity (FNPA) Screening Tool) designed to assess family environmental and behavioral factors that may predispose a child to becoming overweight.

**Methods:**

The FNPA instrument was developed using constructs identified in a comprehensive evidence analysis conducted in collaboration with the American Dietetics Association. Two or three items were created for each of the ten constructs with evidence grades of II or higher. Parents of first grade students from a large urban school district (39 schools) were recruited to complete the FNPA screening tool and provide permission to link results to BMI data obtained from trained nurses in each school. A total of 1085 surveys were completed out of the available sample of 2189 children in the district. Factor analysis was conducted to examine the factor structure of the scale. Mixed model analyses were conducted on the composite FNPA score to determine if patterns in home environments and behaviors matched some of the expected socio-economic (SES) and ethnic patterns in BMI. Correlations among FNPA constructs and other main variables were computed to examine possible associations among the various factors. Finally, logistic regression was used to evaluate the construct validity of the FNPA scale.

**Results:**

Factor analyses revealed the presence of a single factor and this unidimensional structure was supported by the correlation analyses. The correlations among constructs were consistently positive but the total score had higher correlations with child BMI than the other individual constructs. The FNPA scores followed expected demographic patterns with low income families reporting lower (less favorable) scores than moderate or high income families. Children with a total score in the lowest tertile (high risk family environment and behaviors) had an odds ratio (OR) of 1.7 (95% CI = 1.07 – 2.80) compared to children with a total score in the highest tertile (more favorable family environment and behaviors) but this effect was reduced when parent BMI was included as a covariate.

**Conclusion:**

The results support the contention that the FNPA tool captures important elements of the family environment and behaviors that relate to risk for child overweight.

## Background

An '*obesigenic*' environment that contributes to overeating and physical inactivity (i.e., sedentariness) has been implicated as the major contributing factor to the obesity epidemic [1,2]. Environmental factors influence human behaviors in all segments of the population, but the issues are unique among children. Parents directly influence children's physical activity and nutrition behaviors and also dictate the physical and social environments that are available to their children.

Numerous studies have confirmed the importance of parenting behaviors and home environments on children's nutrition and physical activity behaviors [3-7]. While adults can choose to be physically active or inactive and make their own dietary choices, children do not have complete control over these same behaviors. Davison and Birch [4] found that obesigenic environments could be characterized to some degree by parents' physical activity and dietary behavior. More specifically, they found that parents who ate poorly and participated in the least amount of physical activity were more likely to be overweight and also have overweight daughters. While genetic factors clearly have a strong influence on risk, family environments may compound and exacerbate these risks. Despite good intentions, some families may unknowingly create an unhealthy (obesigenic) environment that could predispose their children to becoming overweight.

The development of screening tools has been recommended by many public health groups and agencies in an effort to identify youth that are at an increased risk of overweight [8-10]. Early detection and intervention is considered critical because obesity is known to track quite well through the lifespan [11] and because treatment is more effective at earlier stages [10]. The predominant "*prevention*" paradigm used in clinical settings involves evaluating one or both parents' weight and/or screening to detect children that are "at risk" of overweight (e.g., a body mass index (BMI) between the 85th – 95th age- and sex-specific percentiles) [12]. While this type of screening is important, primary prevention theory would suggest that prevention efforts begin before the child is "at risk". Screening procedures are needed to evaluate home environments and behaviors that increase the child's likelihood of becoming overweight. These factors could then be targeted in follow-up counseling to help prevent the development of overweight in youth. The Institute of Medicine [8] made the following specific recommendation: "*Health care professionals should routinely track BMI, offer relevant evidence-based counseling and guidance..."*. Similarly, the American Academy of Pediatrics [10] specifically recommended that "*families should be educated and empowered through anticipatory guidance to recognize the impact they have on their children's development of lifelong habits of physical activity and nutritious eating" *(p. 427).

Despite the importance of effective BMI screening, there are currently no validated screening tools that may identify modifiable family nutrition and physical activity behaviors that predispose children to become overweight or obese. Some materials and tools have been developed to enhance awareness of healthy eating and physical activity but they are based on general principles and are not capable of predicting children's risk of becoming overweight. Research instruments that assess factors influencing diet or physical activity behavior are also available but they are typically lengthy and don't capture the diverse array of family nutrition and physical activity environments and behaviors that may influence risk for obesity [13].

This paper summarizes the development of an easy to use screening tool (The Family Nutrition and Physical Activity (FNPA) Screening Tool) designed to assess family environmental and behavioral factors that may predispose a child to becoming overweight. Physicians and school nurses are ideally positioned to provide counseling and anticipatory guidance to help parents make changes in their family environment and behaviors [1] so emphasis was placed on developing a tool that could be readily used in these settings. The instrument was evaluated in a large urban Midwestern school district by examining cross sectional associations between family environmental factors and behaviors and measured BMI. Descriptive analyses also examined differences in FNPA scores across different ethnic and socio-economic groups to capture variability in home environments, behaviors, and policies in the population.

## Methods

### Survey development and delivery

The development of the FNPA instrument was guided by an ongoing Evidence Analysis (EA) project supported by the American Dietetic Association (ADA) designed to determine the strength of evidence linking overweight or obesity with specific physical activity and diet behaviors [14]. The systematic literature review was conducted by a team of researchers from across the country using the established ADA Evidence Analyses procedures [14,15]. Ten main factors were identified that had positive associations with overweight and obesity and these were used to create ten distinct FNPA constructs [13]. A total of 21 survey questions were created so that all of the constructs could be captured with at least 2 items. The questions were coded on a 2, 3, or 4 point likert scale depending on the number of possible responses to each question. Seven individual items were reverse coded so that higher scores on all items were most favorable. The answer to each question within a construct was then added together to create a total for that construct. All answers were added together to create a total score for the whole screening tool. The total score was then used to interpret the results of the family environment and behaviors. A high total score implies more favorable family environment and behaviors versus a low total score or high risk family environment and behaviors.

The survey was reviewed and refined by members of the ADA EA team, experts in diet and exercise behavior, and survey statisticians. The survey was originally written in English, but a Spanish version of the survey was created to meet the needs of Spanish speaking parents in the school district.

### Subjects

The target population consisted of parents of first grade children from elementary schools in a large Midwest urban school district. The school district conducts annual height and body mass measurements on all first grade students so approval for the study was first obtained from the school district to allow access to student anthropometric data. Nurses from individual schools were invited to participate in the project and received training and equipment support to assist in collecting the anthropometric data. Nurses from a total of 37 of the 39 elementary schools agreed to participate. The sample of first grade children from the participating schools was predominantly white (57.5%), with smaller percentages of African-Americans (15.3%), Hispanics (16.9%), Asians (5.6%), and other minorities (e.g., Native Americans, mixed ethnicities) (4.7%). Schools varied widely in socioeconomic status with 5 schools (n = 121) of high SES status (fewer than 33.33% of the students eligible for free or reduced priced lunches), 17 schools (n = 413) of middle SES status (33.34% to 66.66% of the students eligible for free or reduced lunches), and 15 schools (n = 320) of low SES status (66.67% or more of the students eligible for free or reduced lunches).

### Procedures

Anthropometric data were obtained by trained nurses using a standardized protocol [16]. Nurses received group training by an expert technician (MI) at a district in-service session prior to the start of the school year and through follow up visits to individual schools. After completing the training, nurses were provided with access to an anthropometry kit containing a Lifesource MD Profit scale (Milpitas, CA) and a SECA Road Rod stadiometer (Hanover, MD). Measurements were obtained in a private setting (nurse's office) with students wearing light clothing without shoes. Body mass was measured to the nearest 0.1 kg and height was measured to the nearest 0.25 cm.

After completing the anthropometric measurements, nurses gave children a packet containing information about the study, a copy of the informed consent document and the FNPA survey. Parents were instructed to return the surveys to the classroom teacher or nurse. A total of 2189 surveys were sent home to first grade parents in the fall. A total of 941 surveys were returned after this initial request (return rate of 43.0%). Several nurses requested a Spanish survey, so a total of 172 translated Spanish surveys were distributed at 9 of the schools. A total of 18 Spanish surveys were returned for a 10.5% return rate. A follow up survey was also distributed (later in the fall) by 21 of the nurses to give parents a second chance to participate. A total of 691 follow-surveys were sent home and 126 were returned for a return rate of 18.2%. The final number of completed surveys was 1085 for a final return rate of 49.6%. The gender of the survey respondent was not asked. The overall procedures were approved by the Institutional Review Board at the University.

### Data processing

The nurses provided investigators with height and body mass data using student ID numbers (without names) to ensure confidentiality. Key demographic information such as child gender, birthdate and ethnicity were obtained from the school district and merged with the BMI data using the common district ID number. Individual BMI values were calculated according to standard convention [weight (kg)/(height (m))^2^]. Height, body mass and BMI percentiles were computed for each child using the SAS growth chart programs available on-line from the CDC . The computed BMI percentiles were then used to categorize children as normal weight (< 85th percentile), '*at risk for overweight*' (between the 85th and 95th percentiles) and '*overweight*' (at or above the 95th percentile), according to standard CDC definitions [17].

The FNPA survey data was entered into Microsoft Excel, manually checked for accuracy by an independent data entry analyst, and then scanned for completeness of the records. The final FNPA data were coded with the student ID and merged with the child BMI data. A small number of surveys (n = 55) were eliminated from analyses because they lacked some critical data. Another 176 surveys were eliminated because one or more of the 21 survey questions were not answered. Therefore, the final sample size for the present study was 854.

### Data analyses

Descriptive statistics and prevalence of '*at risk for overweight*' and '*overweight*' for the children were calculated for both the participating and non-participating students in order to determine whether the participating sample was representative of the overall population.

The preliminary analysis focused on the psychometric properties of the FNPA tool. Factor analyses were conducted using varimax rotation to examine the factor structure of the FNPA instrument and the fit of individual items. Internal consistency of the items was also evaluated with alpha reliability.

Construct validity was examined with a variety of analyses. Descriptive analyses were performed to examine differences in FNPA scores within the sample population. Mixed model analysis of variance controlling for the nested nature of the data (children aggregated into different schools) was used to test possible differences in weight status and FNPA scores by ethnicity and socioeconomic status. The Tukey-Kramer post-hoc test was used to test for group differences on individual construct scores. These mixed model analyses were performed using Proc Mixed routines in SAS version 9.0 (Cary, NC). Correlations among FNPA constructs and other main variables were computed to examine possible associations among the various factors. The presence of significant correlations among the variables would suggest that they co-vary and may be indicative (or characteristic) of an overall "obesigenic" environment. Finally, logistic regression was used to examine the construct utility of the FNPA total score with the likelihood of being overweight. Two dummy variables were used to code the three risk categories based on the FNPA total score. The referent category was the low risk or high FNPA total score tertile. The descriptive, correlation and logistic regression analyses were performed using Statistical Package for Social Sciences (SPSS) statistical software version 14.0 (Chicago, IL).

## Results

There were similar numbers of males (n = 438) and females (n = 416) in the final sample. The percent of subjects from each racial/ethnic group were as follows: 68.0% Caucasian, 11.6% African American, 11.5% Hispanic, and 4.8% Asian, (4.1% were classified as "Other). The mean BMI for males was 17.4 kg/m^2 ^and the mean BMI for females was 17.1 kg/m^2^. Based on CDC classifications, 18.5% of males and 16.1% of females were categorized as '*at risk for overweight*' while 18.0% of males and 16.1% of females were categorized as '*overweight*'. These classifications were not significantly different (p = 0.96) from the total sample of school children from which this cohort was taken. In the total sample, 36.4% of males and 35.3% of females were either *'at risk for overweight' *or *'overweight' *[18].

Demographic data from the parent survey suggested that the sample population was reasonably diverse with regard to income and ethnicity. Approximately 44.2% of the mothers had a high school education or less while 55.1% of the fathers had a high school education or less. Family income was reported as follows: 34.3% earned less than $25,000, 33.2% earned between $25,000–$50,000, 17.9% earned between $51,000–$75,000, and 14.6% earned greater than $75,000. The mothers' mean BMI was 26.9 kg/m^2 ^and the fathers' mean BMI was 27.5 kg/m^2^. Less than half (45.1%) of the mothers and only 31.7% of the fathers had normal BMI values (less than 25.0 kg/m^2^).

### Psychometric results

Factor analyses were conducted to examine the psychometric properties of the FNPA tool. Seven factors met the minimum eigenvalue criteria of 1.0 but the scree plot indicated a clear separation between the first factor (eigenvalue of 3.32) and the other factors (average eigenvalue of 1.25). Fourteen of the 21 items loaded significantly on the first factor (loadings ranged from 0.30 to 0.58) with 3 other items approaching significance (See Table 1). Two questions (Q2 and Q3) were found to have low (0.13) or negative (-0.29) loadings. The items comprised the "restriction and reward" construct but the wording may have been confusing to participants. Parents may have perceived restriction of access to snack foods as a good dietary practice when; in fact, it was intended to reflect a negative or overly restrictive home environment. The literature shows restriction to be negatively correlated with measured BMI [19], but the negative loadings indicate that responses were not consistent with other items on the survey. Because these items were not interpreted correctly by the participants, they were removed from the dataset.

**Table 1:** Factor analysis loadings for Family Nutrition and Physical Activity Survey

**Variable**	**Factor 1**	**Factor 2**	**Factor 3**	**Factor 4**	**Factor 5**
Q1	.41	35			

Q 4			36		

Q 5	.31		42		

Q 6				.65	

Q 7				.62	

Q 8		47			.55

Q 9	.30				-.53

Q 10	.35		38		

Q 11	.45				

Q 12	.46	31			

Q 13	.58				

Q 14	.40		-.50		

Q 15	.57		-.47		

Q 16	.45	.39			

Q 17	.47				

Q 18	.53	-.51			

Q 19	.53	-.49			31

Q 20	.51	-.53			

Q 21			.53		

Eigenvalue	3.24	1.59	1.46	1.26	1.08

% Variance	17.1	8.4	7.7	6.6	5.7

Cumulative % Variance	17.1	25.5	33.2	39.8	45.5

Another factor analysis was performed to see if this influenced the factor structure. There were 6 factors with eigenvalues greater than 1.0 but only the first 5 were interpretable (see Table 1). All of the items loaded positively on Factor 1 with loadings ranging from .19 to .58. Factor 2 was characterized by a positive loading on a television viewing question (Does your child have a TV in their bedroom?) and negative loadings on three physical activity questions that also loaded significantly on Factor 1. Factor 3 was characterized by significant negative associations on two diet questions related to consumption of fast food and opposite associations on sleep (Q5) and amount of sports participation (Q21). This pattern suggests that involvement in sports was somewhat associated with a tendency to prepare fast, simple meals. The diet items (Q14 and Q15) also loaded significantly on Factor 1 so the relevance of this factor is not clear. Factor 4 was characterized with significant associations on questions about TV viewing (Q6) and video game usage (Q7) while Factor 5 was characterized by significant loadings on two questions related to TV (the presence of TV in the bedroom (Q8) and parental monitoring of TV [9]). These items did not load significantly on Factor 1 but positive loadings were evident suggesting that there was some association with the other items. The resulting pattern suggests that TV behaviors and policies about sedentary behavior may be somewhat independent from the other items on the instrument but the high eigenvalue, scree plot distribution and consistent associations on Factor 1 support the notion that the items are part of a common underlying construct. The alpha reliability of the single factor FNPA scale (0.72) also indicates that there is reasonable internal consistency in the FNPA instrument. The alpha reliability with all 21 items was only slightly lower (alpha = 0.70) but the remaining analyses were conducted without the two items on restriction/reward.

### Descriptive results – population comparisons

The composite FNPA total score was evaluated to examine possible associations with family socio-economic status/income and ethnicity (Table 2). When stratified by income, significant differences were seen for the construct total score (p < .05). In general, higher income families had higher, or more favorable, scores. When stratified by ethnicity, significant differences were seen for the construct total score (p < .05). In general, Caucasian families had higher, or more favorable, scores compared to the other ethnicities.

**Table 2:** FNPA total score by ethnicity and income

**Ethnicity**	**Construct Total Score**
Caucasian (n = 581)	51.89(5.3)

African-American (n = 99)	50.35(5.0)†

Hispanic (n = 98)	49.96(4.8)†

Asian (n = 41)	48.32(4.8)†‡

Other (n = 35)	49.97(5.1)†

**Income**	

< $25,000 (n = 249)	49.84(5.1)

$25,000–$50,000 (n = 276)	51.27(4.8) †

$50,001–$75,000 (n = 159)	51.30(5.3) †

> $75,000 (n = 130)	53.80(5.4) †‡*

Values are mean (SD); † significantly different from Caucasian;

‡ significantly different from African-American;

* significantly different from Hispanic;

§significantly different from Asian

† significantly different from < $25,000;

‡ significantly different from $25,000–$50,000;

* significantly different from $50,001–$75,000

**Figure 1:**
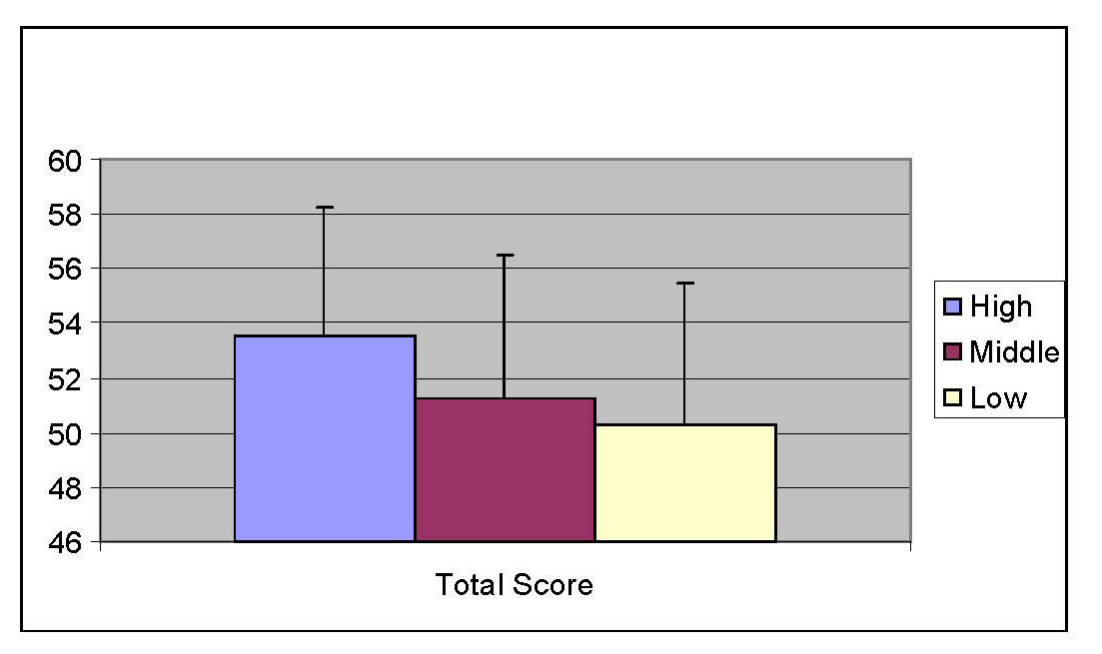
**Total survey score by school**.

To examine the impact of SES in more detail, school level differences in FNPA total scores were compared (See Figure 1). In general, high SES schools had higher, or more favorable, scores than lower SES schools. The high SES schools were significantly different from the middle and low SES schools for the construct total score (p < .05). The middle SES schools were also significantly different from the low SES schools for the construct total score (p < .05).

### Correlation results

Correlations among the main outcome variable of child's BMI, and parents BMI and the FNPA constructs are shown in Table 3. There were significant correlations (p < 0.01) between child's BMI and mother's BMI, father's BMI, school SES, and the constructs of breakfast/family meal, model nutrition, high calorie beverages, TV in the bedroom, child's physical activity, and total score. There were also significant correlations (p < 0.05) between child's BMI and parent's physical activity and sleep schedule. Most of the constructs were significantly correlated (p < 0.05) with each other with correlations ranging from 0.07 to 0.39. Each construct was also significantly correlated (p < 0.01) with the total score with correlations ranging from 0.32 to 0.66.

**Table 3:** Correlation matrix: child's BMI with constructs

	MBMI	FBMI	Sch SES	BrkFam	ModNut	NutDns	HiCalBev	ScrTime	TVBed	ParentPA	Child PA	Sleep Sch	TotalScr
Child's BMI	.267**	.155**	.173**	-.094**	-.132**	-.051	-.129**	-.020	-.156**	-.086*	-.111**	-.080*	-.173**
Mother's BMI	1	.225**	.136**	.007	-.125**	-.073*	-.129**	-.098**	-.072*	-.175**	-.133**	-.049	-.184**
Father's BMI		1	.018	-.058	-.128**	-.093*	-.121**	-.171**	-.090*	-.091*	-.011	-.070	-.163**
School SES			1	-.093**	-.124**	-.037	-.123**	-.029	-.198**	-.049	-.214**	-.195**	-.205**
Breakfast/Family Meal				1	.215**	.320**	.202**	.049	.126**	.217**	.188**	.162**	.532**
Model Nutrition					1	.341**	.274**	.197**	.266**	.170**	.099**	.182**	.535**
Nutrient Dense Foods						1	.263**	.133**	.150**	.259**	.206**	.116**	.658**
High Calorie Beverages							1	.146**	.257**	.191**	.215**	.189**	.606**
Screen Time								1	.141**	.113**	.019	-.002	.317**
TV in Bedroom									1	.062	.079*	.209**	.386**
Parent's Model PA										1	.391**	.069*	.606**
Child's PA											1	.189**	.565**
Sleep Schedule												1	.387**

Seven of the 21 individual items that make up the 10 constructs were reverse coded so that higher scores on all items were most favorable.

### Logistic regression results

Logistic regression was used to evaluate the construct validity of the instrument. The total score from all of the respondents was divided into tertiles to test for differences in BMI between families with high total scores (more favorable family environment and behaviors) versus those of middle (moderate family environmental and behavioral risk) or low total scores (high risk family environment and behaviors). The total score significantly predicted an increased risk for being *'at risk of overweight' *or *'overweight' *(above the 85^th ^percentile for BMI) in these children in the high risk group (p = 0.026). Children with a total score in the lowest tertile (higher risk family environment and behaviors) had an odds ratio (OR) of 1.7 (95% CI = 1.07 – 2.80) compared to children with a total score in the highest tertile (lower risk family environment and behaviors) for being *'at risk of overweight' *or *'overweight'*.

Because school SES, family income, and parent BMI may influence a child's environment and behaviors we conducted additional logistic regression using school SES, family income, and parent BMI as covariates. The inclusion of school level SES as a covariate did not appreciably alter the OR value or the confidence intervals. The inclusion of parent income as a covariate reduced the OR suggesting that it explains some of the effect on the family environmental and behavioral variables. While the results just barely fell below the value of 1.0 for the lower bound of the confidence interval, the OR value still suggested a noteworthy effect. When parent BMI was included in the analyses, the OR values were no longer significant. These findings suggest that parent BMI and parent income need to be considered when examining results from the FNPA.

## Discussion

The study provides descriptive information and preliminary validation for a comprehensive screening tool designed to assess modifiable home environments, practices, and behaviors that are associated with risk of childhood overweight. A variety of cross-sectional comparisons were made between the FNPA constructs and measured BMI to examine construct validity of the tool. The sample population was representative of parents (and children) in the entire district with regard to BMI and ethnic distribution. The percentage of *'at risk for overweight' *and *'overweight' *children in this district is typical of the patterns in children throughout the United States as 36.5% of males and 32.2% of females were *'at risk for overweight' *and *'overweight'*[11,21-23].

Mixed model analyses revealed the expected patterns in BMI and family environment and behaviors. Generally, children from higher income families had higher, or more favorable, total scores. Caucasians also had significantly higher, or more favorable, total scores than the other ethnicities. Children from high SES schools had higher, or more favorable, total scores than children from lower SES schools. These results fit with the observed patterns in BMI with children from higher income families (> $50,000), Caucasians children, and children from high SES schools having lower mean BMI values. In general, these higher SES schools are predominately Caucasian (85.1% for high SES schools and 73.6% for middle SES schools), so the observed patterns in construct scores and BMI would be expected. These results are consistent with previous studies [20,21]. In a nationally representative sample of children, Hispanic and African-American female children were more likely to be overweight when compared to Caucasian female children as well as Hispanic male children when compared to their male Caucasian counterparts [22]. A study of New York City school children also found that Hispanic children were more likely to be significantly overweight when compared to Caucasian and other ethnic groups [23]. The fact that these similar demographic relationships were evident in the comparison of the FNPA scores provides support for the construct validity of the tool.

The significant correlations between child and parental BMI confirms previous studies [24,25]. Although the correlation cannot distinguish between genetic and shared family environment and behaviors, it does point out that the predominant factor influencing child BMI is parental BMI. However, significant correlations between BMI and most of the FNPA constructs (breakfast/family meal, model nutrition, high calorie beverages, TV in the bedroom, parent physical activity, child physical activity, sleep schedule, and overall construct score) were also observed. This suggests that the constructs assessed in the FNPA tool capture aspects of the home/shared family environment and behaviors that also influence child BMI. It is noteworthy that the overall construct score had higher correlations with child's BMI (-.17) than any of the individual items. This finding highlights the importance of capturing a diverse array of family environments and behaviors when trying to assess child risk for overweight.

The correlations among constructs were generally low to moderate (range: 0.07 to 0.66 with the average pairwise correlation coefficient of 0.24). The consistently positive correlations among constructs indicate that there is a tendency for family-based obesigenic behaviors to cluster to some extent. Families that indicated negative behaviors/settings were more likely to report other less desirable characteristics while families with more favorable scores were likely to report other favorable characteristics. The tendency for behaviors to cluster together in this way supports the notion that home environments and behaviors can be characterized as more or less '*obesigenic*'. The inherent goal of the FNPA screening tool is to provide a quick way to characterize and score these modifiable family environments and behaviors so that changes can be implemented to reduce risks for overweight.

Logistic regression analyses were conducted to further evaluate the construct validity of this tool. The analyses examined whether the composite summary variable was related to increased likelihood of being *'at risk for overweight' *or *'overweight'*. The results showed that the total score significantly predicted an increased risk for being *'at risk of overweight' *or *'overweight' *(above the 85^th ^percentile for BMI; p = 0.026). Additional analyses were conducted to control for school and family SES as well as parent BMI. These values were used as covariates in the logistic regression. The results indicated that after controlling for school SES, children with a low FNPA total score were still at significant risk for being *'at risk of overweight' *or *'overweight'*. However after controlling for family income, the OR for the total FNPA score for these high risk children did not change but did fall just below significance. The total FNPA score also lost significance when parent BMI was included. These results are not surprising considering the documented importance of parent weight status on a child's risk for being or becoming overweight. While genetics may explain some of this effect it is also possible that aspects of the home environment and behaviors may differ in families with higher levels of BMI. The cross sectional nature of the study does not allow a direct evaluation of the predictive utility of the FNPA tool so the focus at this stage was to examine the construct validity of the instrument.

To our knowledge, this is the first tool that combines information from a variety of behaviors (e.g. diet, physical activity and inactivity, sleep, family structure) into a single behaviorally-based obesity screening tool. A previous study by Golan and Weizman [26] reported on results from a tool called the Family Eating and Activity Habits Questionnaire which assessed eating patterns of the family as well as family rules regarding eating behaviors. However, the main difference between this tool and the one descried herein is that the Family Eating and Activity Habits Questionnaire was mainly developed to assess family environmental changes associated with weight loss in obese children, whereas our tool is aimed at preventing obesity. Their instrument included four scales: physical activity level, stimulus exposure, eating related to hunger, and eating style. These four scales mainly focused on physical activity and inactivity of the parents and child, the types of food in the house, eating and hunger, and what the child was doing while they were eating. They found that the total family score on the questionnaire was significantly related to the child's weight status. Correlations among the four scales and child's weight loss ranged from 0.36 and 0.73. The FNPA survey is a different tool in that it assesses a child's risk for becoming overweight prior to the actual weight gain. Unlike the Family Eating and Activity Habits Questionnaire, the FNPA survey also includes items assessing other variables associated with risk for overweight (e.g. sleep, screen time habits, TV in the bedroom, and family schedule and rules).

Other studies have investigated links between specific behaviors and BMI or parent related behaviors on childhood behaviors [27-30]. Saelens et al [30] investigated the home environment as it related to screen time and found a relationship between TV watching and children's weight status in early childhood as well as overweight status in older children who watched more than 2 hours of TV per day. Campbell et al [28] utilized several lengthy surveys to assess a family's food environment including: perception of child's diet, modeling of eating and feeding strategies, availability and preference of foods, confidence in cooking and mealtime interruptions, and TV viewing. They found that several of these aspects were associated with certain dietary outcomes that may be associated with fatness. Arrendondo and colleagues [27] investigated parenting styles and its influence on obesity in children, and found that healthy eating and exercise habits in children were associated with positive reinforcement, monitoring, and appropriate discipline from the parents. Finally, Salmon et al [29] investigated the family environment as it relates to TV viewing and physical activity through both a parent and child survey, and found that SES, frequency of TV viewing by the family, parents TV viewing habits, and rules for TV viewing during meals were associated with the amount of TV the child watched.

In contrast to previous studies we used a holistic approach that captures the full constellation of family environmental behaviors that relate to a child's risk of becoming overweight. As described above, most previous studies investigated one or two aspects of the home environment and/or behaviors, and many of these studies utilized lengthy and time-consuming surveys. The FNPA tool was based on constructs that were shown in a comprehensive Evidence Analyses to predict child weight status. It incorporates family behaviors and practices related to diet, screen time, physical activity (of both the parents and child) as well as family rules, family meals and sleep schedules. Several pieces of evidence support the contention that the instrument captures overall family obesigenic environment and behaviors. The factor analyses indicated that the items loaded on a single factor. The correlation analyses indicated that scores on individual constructs tended to be associated with each other. Finally, stronger correlations were observed between total FNPA score and BMI indicating that this composite score was more related to risk for high BMI.

Some strengths of this study include the large representative nature of the study (involvement from 37 of 39 schools in the district) and the investigation of multiple family environmental factors and behaviors that may influence a child's risk for becoming overweight as well as the integration of family and school demographic factors. A clear limitation of this study is the cross sectional nature of the data. Many factors can influence a child's current weight status, and the cross sectional nature of this study does not allow for investigation of what may have caused these children to be overweight. We contend that modifiable, environmental and behavioral factors may exert an independent influence on children's risk for becoming overweight. Genetic and family factors may establish an underlying risk but it is likely that home environments and behaviors would either moderate or exacerbate this potential risk as the child grows and matures. With longitudinal data it would be possible to evaluate the ability of the FNPA tool to predict change in BMI over time (this work is currently in progress). Longitudinal analyses would also help to examine the relative importance of individual constructs on predicting risk. This would make it possible to create a weighted risk score in which the overall FNPA score reflects the combination of the family behavior and associated risks. The present results support the construct validity and potential utility of the FNPA tool for further study of these relationships.

## Conclusion

The results support the contention that the FNPA survey captures important elements of the family environment and behaviors that relate to risk for child overweight. The FNPA survey has potential for use by obesity researchers as well as by a variety of clinical and public health professionals (e.g. pediatricians and school nurses) for a quick-and-easy manner of assessing a child's home environment and behaviors and their risk for becoming overweight. The survey could be an invaluable tool for providing individualized feedback and intervention information to families about changes that can be made in their home environment and behaviors.

Future research is needed to investigate the use of the FNPA survey in a longitudinal sample of children as well as testing a revised version of the FNPA survey that will utilize a more clinical friendly version that has resulted from these analyses as well as feedback from parental focus groups.

## Competing interests

The authors declare that they have no competing interests.

## Authors' contributions

MI helped design the study, carried out all data collection, performed analyses, and drafted the manuscript. GW contributed to the conception and design of the study, helped with the analyses and interpretation of the data, and helped revise the manuscript. JE contributed to the design of the study, helped with interpretation of the data and revision of the manuscript. SN contributed to the design of the study, assisted with the analyses and interpretation of the data, and helped revise the manuscript. All authors read and approved the final manuscript.
